# Determination of antiprotozoal drug mechanisms by metabolomics approaches

**DOI:** 10.1017/S0031182013000814

**Published:** 2013-06-05

**Authors:** DARREN J. CREEK, MICHAEL P. BARRETT

**Affiliations:** 1 Department of Biochemistry and Molecular Biology, Bio21 Molecular Science and Biotechnology Institute, University of Melbourne, 30 Flemington Rd, Parkville, Victoria 3010, Australia; 2 Wellcome Trust Centre for Molecular Parasitology, Institute of Infection, Immunity and Inflammation, and Glasgow Polyomics, College of Medical, Veterinary and Life Sciences, University of Glasgow, Glasgow G12 8TA, UK

**Keywords:** metabolomics, mode of action, *Trypanosoma brucei*, protozoan parasite, *Plasmodium falciparum*, antiprotozoals

## Abstract

The discovery, development and optimal utilization of pharmaceuticals can be greatly enhanced by knowledge of their modes of action. However, many drugs currently on the market act by unknown mechanisms. Untargeted metabolomics offers the potential to discover modes of action for drugs that perturb cellular metabolism. Development of high resolution LC-MS methods and improved data analysis software now allows rapid detection of drug-induced changes to cellular metabolism in an untargeted manner. Several studies have demonstrated the ability of untargeted metabolomics to provide unbiased target discovery for antimicrobial drugs, in particular for antiprotozoal agents. Furthermore, the utilization of targeted metabolomics techniques has enabled validation of existing hypotheses regarding antiprotozoal drug mechanisms. Metabolomics approaches are likely to assist with optimization of new drug candidates by identification of drug targets, and by allowing detailed characterization of modes of action and resistance of existing and novel antiprotozoal drugs.

## INTRODUCTION

Protozoan parasites, including *Plasmodium* spp., *Trypanosoma* spp. and *Leishmania* spp., are responsible for diseases that cause significant morbidity, mortality and economic burden, predominantly in developing countries. There are currently no effective vaccines available for the prevention of these tropical diseases, including malaria, human African trypanosomiasis (HAT), Chagas disease and leishmaniasis, and therapy relies heavily on antiprotozoal drugs (Pink *et al.* 2005; Anthony *et al.* 2012; Barrett and Croft, 2012). New antiprotozoals are urgently required, as most existing drugs suffer from one or more liabilities related to poor efficacy, drug resistance, toxicity, high cost or unsuitable pharmacokinetic properties (Wells *et al.* 2009; Phillips, 2012).

The lack of suitable treatments for these tropical diseases is partly due to there having been inadequate pharmaceutical research efforts during the last century (Renslo and McKerrow, 2006; Wells *et al.* 2009). The majority of current antiprotozoal drugs did not arise from programmes common in contemporary pharmaceutical development, and the rigorous pre-clinical and clinical evaluation that is now expected of new drugs did not accompany registrations of most of the antiprotozoals (Renslo and McKerrow, 2006). Indeed, many commonly used antiprotozoal drugs act by unknown mechanisms, which severely impedes optimal clinical utilization and monitoring for efficacy, toxicity and resistance.

Fortunately, the pipeline for new antiprotozoal drugs now looks promising as a result of recent efforts to screen large compound libraries against these pathogenic parasites. Hundreds of ‘hit’ compounds have been identified that inhibit the *in vitro* proliferation of specific protozoan parasites (Gamo *et al.* 2010; Guiguemde *et al.* 2010; Duffy and Avery, 2012; Sykes *et al.* 2012). It is clear that screening compound libraries against organisms is superior to primary screens against individual targets, possibly due to the fact that key pharmacological criteria such as membrane-permeability are already built into compounds passing the former type of screen. A major bottleneck in the hit-to-lead optimization pipeline following identification of hits in whole organism screens relates to the lack of mechanistic information concerning their modes of action (Guiguemde *et al.* 2012). Identification of target(s) for hit compounds allows rational medicinal chemistry to improve selectivity of binding to the parasite target, and ensures that structural modifications to enhance the pharmacokinetic and toxicity profiles are not likely to compromise activity. The availability of methods to identify modes of action for antiprotozoal compounds in an untargeted manner would greatly enhance the efficiency of drug discovery for parasitic diseases.

Metabolomics is an emerging technology that provides an untargeted overview of cellular metabolism by the simultaneous detection and relative quantification of hundreds of small molecules (<1500 Da) in a biological system (Scalbert *et al.* 2009; Scheltema *et al.* 2010; Creek *et al.* 2012*a*; Dunn *et al.* 2012). Metabolomic profiling of protozoan parasites treated with antiprotozoal compounds can detect drug-induced changes to parasite metabolism and identify the individual metabolites and pathways that are directly perturbed. This provides a rapid and unbiased method to discover likely drug targets (Beyoğlu and Idle, 2013).

The untargeted nature of metabolomics has allowed rapid and unbiased classification of numerous antimicrobial compounds according to their modes of action (Gao *et al.* 2007; Liu *et al.* 2009; Halouska *et al.* 2012). Investigation of the metabolic response of *Staphylococcus aureus* to triphenylbismuthdichloride revealed pyruvate dehydrogenase as a target for this novel antibiotic (Birkenstock *et al.* 2012). A more detailed elucidation of the mechanism of antibacterial action was demonstrated by isotope-labelled metabolic flux profiling of *Escherichia coli* following antifolate exposure (Kwon *et al.* 2008). Trimethoprim is a known inhibitor of dihydrofolate reductase (DHFR), which was confirmed by accumulation of oxidized folates in the treated cells. However, an unexpected inhibiton of folylpoly-gamma-glutamate-synthetase was also observed; a response to dihydrofolate accumulation (Kwon *et al.* 2008). This systems-based approach allows observation of pharmacological effects that often go unnoticed in classical biochemical or genetic approaches based on individual candidate drug targets. Here we discuss the development of platforms that have enabled measurement of hundreds of metabolites in protozoa and their application to drug-target identification.

## METABOLOMICS METHODOLOGY

Metabolomics studies fall into two broad categories, targeted and untargeted. Targeted studies involve hypothesis-driven experiments that aim to provide accurate quantification of a subset of known metabolites, usually restricted to a particular metabolic pathway or chemical class. Untargeted studies by contrast are usually hypothesis-generating experiments that provide relative quantification of all detectable metabolite signals, with subsequent identification of the most significant metabolites (Dunn *et al.* 2012). Untargeted studies are particularly attractive for the investigation of drugs of unknown mode of action. Targeted studies inherently provide less coverage of the metabolome, but have the advantage of enabling accurate quantification as well as flux analysis to determine dynamic responses in candidate pathways (Kwon *et al.* 2008).

### Sample preparation

**Fig. 1.**
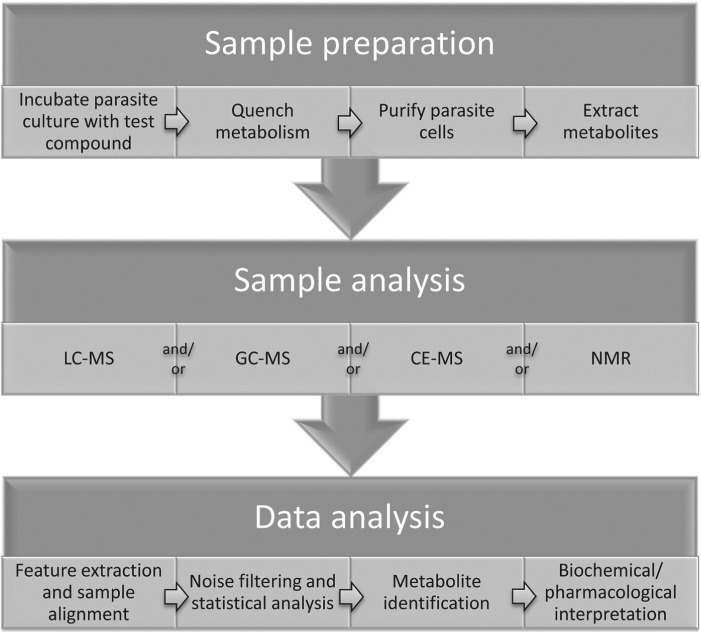
General outline of methodology for metabolomics studies of protozoan parasites in cell culture.

Measurements of metabolic responses to drugs are often obtained from *in vitro* cell culture systems (Fig. 1). This approach allows maximum control over genetic and environmental variables, such as temperature, pH, atmosphere, extracellular environment and cell density, which can contribute to unwanted metabolic alterations and obscure the desired observation of drug-specific effects (Cuperlovic-Culf *et al.* 2010). The choice of culture media may interfere with drug action and/or metabolic responses, particularly when a standard culture medium does not mimic the *in vivo* environment of the parasite. For example, the high folate levels present in standard *Trypanosoma brucei* culture medium, HMI11 (Hirumi and Hirumi, 1989), inhibits the *in vitro* activity of antifolate compounds that demonstrate trypanocidal activity *in vivo* (Sienkiewicz *et al*. 2008), suggesting that culture media should be modified to more closely reflect *in vivo* conditions wherever possible. In addition, the impact of drug treatment on parasite growth and viability can often alter metabolite abundances, independent of the molecular target of the drug. Multiple controls, time courses and drug concentrations are often necessary to delineate mechanism-related metabolic alterations from non-specific stress responses (Vincent *et al.* 2012).

Intracellular parasites present unique challenges, and isolation of parasites from uninfected host cells should be performed to avoid interference from host cell metabolites (e.g. saponin lysis or MACS purification for *Plasmodium falciparum*) (Zhang *et al.* 2011; Biagini *et al.* 2012), although it is intriguing to consider how impacts on host cell metabolism can contribute to the pharmacological effect of a drug. For example, human cytomegalovirus was shown to lead to up-regulation of host cell fatty acid biosynthesis and inhibitors of fatty acid biosynthesis could inhibit viral growth (Munger *et al.* 2008). Metabolomic analysis of uninfected host-cell response to drug treatment is also recommended to confirm the parasite-specificity of drug-induced metabolic changes. Care must be taken to avoid metabolic perturbation during any purification step, and metabolic quenching is often achieved by rapid cooling to 0 °C (Saunders *et al.* 2011). Following quenching and isolation of cells, metabolites are generally extracted with organic solvent mixtures such as chloroform/methanol/water (1:3:1) (Scheltema *et al.* 2010; t'Kindt *et al.* 2010*b*).

### Sample analysis

Metabolomics analysis is generally performed with mass spectrometry or NMR spectroscopy. Mass spectrometry is usually coupled to chromatographic separation such as gas chromatography (GC-MS), capillary electrophoresis (CE-MS) or liquid chromatography (LC-MS) (Dunn *et al.* 2005; Scalbert *et al.* 2009; Scheltema *et al.* 2010; Creek *et al.* 2012*a*).

LC-MS with high resolution mass spectrometry is becoming the most widely used metabolomics platform due to its high sensitivity, broad specificity and the ability to annotate or identify hundreds of polar and non-polar metabolites from a wide range of metabolic classes (Theodoridis *et al.* 2012). Reversed phase chromatography is well established, and is particularly suitable for analysis of lipids and other non-polar metabolites (Theodoridis *et al.* 2012). Hydrophilic interaction chromatography (HILIC) is finding increasing application in metabolomics, with particular advantages for the separation and identification of polar metabolites (Cubbon *et al.* 2009; Zhang *et al.* 2012). High resolution mass spectrometry is critical for the detection and identification of metabolites in untargeted studies. Recent advances in mass spectrometry have enabled widespread application of untargeted metabolomics, as most new time-of-flight (TOF) and Fourier transform (Orbitrap and FT-ICR) mass spectrometers routinely achieve the required resolution (>30 000) and mass accuracy (<5 ppm) (Dunn *et al.* 2012; Theodoridis *et al.* 2012).

### Data analysis

Computational approaches to metabolomics data analysis are essential, as modern metabolomics platforms routinely detect tens of thousands of ion signals per sample. Many detected peaks arise from noise, contaminants and LC-MS artefacts, and extensive data processing is necessary to allow meaningful interpretation of results (Jankevics *et al.* 2011; Kuhl *et al.* 2011; Creek *et al.* 2012*b*; Weber *et al.* 2012). Numerous open-source software packages are available to provide high-throughput detection, alignment, quantification, filtering and/or identification of metabolite signals, including XCMS, Metalign, MZmine, mzMatch and IDEOM (Smith *et al.* 2006; Lommen, 2009; Pluskal *et al.* 2010; Scheltema *et al.* 2011; Creek *et al.* 2012*b*). Pre-processed metabolomics data provide relative metabolite abundances for hundreds to thousands of putative metabolites for interrogation by univariate or multivariate statistics (Liland, 2011; Vinaixa *et al.* 2012).

The major bottleneck for interpretation of untargeted metabolomics data is metabolite identification (Dunn *et al.* 2012). Many metabolites can be putatively annotated by matching accurate masses to the exact mass of metabolites in biochemical databases. However, accurate metabolite identification is often hampered by metabolites with the same (isomers) or similar (within the error margin of the MS) mass (Scheltema *et al.* 2010). Confirmation of identification requires orthogonal data such as fragmentation spectra (MS/MS) and chromatographic retention time, and should be compared to authentic standards (Sumner *et al.* 2007). Unfortunately, many metabolite standards are not easily accessible, and detected metabolites are often reported as putative, or unknown, identities. Isolation and characterization of unidentified metabolites is labour intensive, and is generally only performed for a few metabolites that show statistical significance in a particular study.

Advanced biochemical interpretation of metabolomics studies can be achieved by software solutions that allow mapping of metabolite levels on to metabolic pathways (Paley and Karp, 2006; Jourdan *et al.* 2010; Leader *et al.* 2011; Yamada *et al.* 2011). The bottlenecks associated with metabolite identification limit the usefulness of these approaches, and pathway-based analyses are best suited to targeted metabolomics studies. A compromise approach, which combines untargeted metabolomics with retention time data from authentic standards to improve the confidence of putative metabolite identifications (Creek *et al.* 2011), is supported by the IDEOM software (Creek *et al.* 2012*b*). Interpretation of data in the context of metabolic pathways may also improve the accuracy of metabolite identification (Rogers *et al.* 2009; Weber and Viant, 2010).

## METABOLOMICS FOR INVESTIGATING THE MODE OF ACTION OF ANTIPROTOZOAL DRUGS

### Trypanocidal compounds

*T. brucei* is the causative agent of HAT, otherwise known as sleeping sickness. Infection occurs following the bite of a Tsetse fly, and parasites remain in the bloodstream during stage 1 disease, where symptoms are minor and non-specific (Barrett *et al.* 2007). Stage 2 disease occurs after parasites enter the central nervous system, leading to neurological complications including disturbed sleep-wake patterns. The infection is inevitably fatal if untreated. Five compounds are approved for the treatment of HAT, but each has serious limitations (Brun *et al.* 2011; Barrett and Croft, 2012). Suramin and pentamidine must be given by injection and are only effective during stage 1 disease because they do not penetrate the blood-brain barrier. Melarsoprol is active against both stage 1 and stage 2 disease, but this arsenical compound is particularly toxic, with 5–10% of patients suffering from reactive encephalopathy, which is often fatal. Eflornithine alone, or in combination with nifurtimox is also active against stage 2 CNS disease (Barrett *et al.* 2007; Phillips, 2012). Clinical utilization of eflornithine may be limited by the requirement for administration of large doses by intravenous infusion, and there is concern that resistance to these drugs may develop rapidly (Vincent *et al.* 2010). Eflornithine is the only compound among the five approved drugs that has a well-defined mode of action, which involves inhibition of ornithine decarboxylase (ODC) and subsequent depletion of polyamines (Grishin *et al.* 1999).

**Fig. 2.**
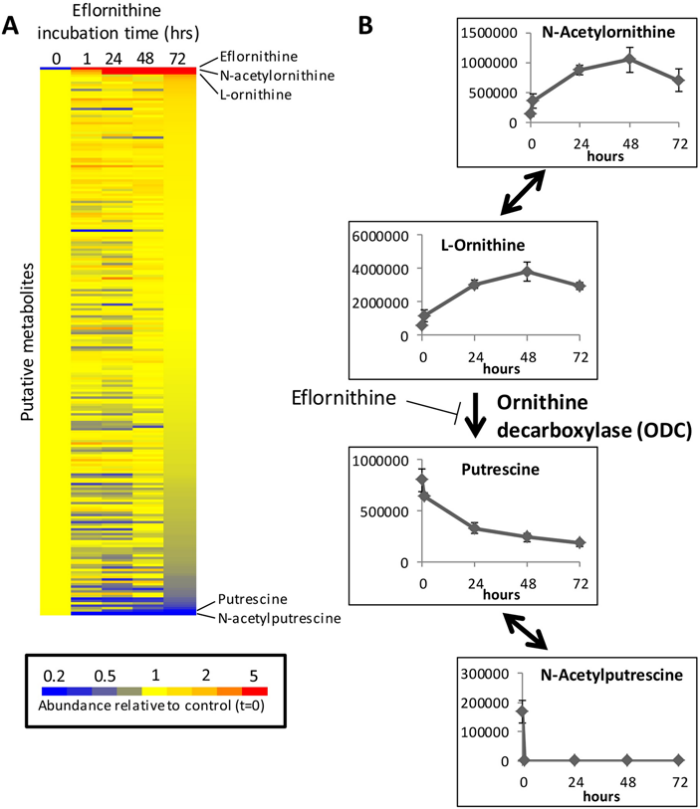
Metabolomic response of *Trypanosoma brucei* to eflornithine over a 72 h incubation time-course (Vincent *et al.* 2012). (A) Heat map shows the relative change in abundance of all putative metabolites following incubation with eflornithine. Putative metabolites are ranked according to fold-change at 72 h. The most significant increases and decreases correspond to metabolites of the polyamine pathway. (B) Metabolite abundance profiles of polyamine pathway metabolites showing accumulation of metabolites upstream of ornithine decarboxylase (ODC), and depletion of downstream metabolites. Y-axes represent peak heights from LC-MS data.

The mode of action of eflornithine was recently confirmed using untargeted metabolomics, providing a proof of concept that untargeted metabolomic profiling of protozoan cell cultures is a powerful tool for the unbiased determination of mode of action for antiprotozoal compounds (Vincent *et al.* 2012). Eflornithine induced significant accumulation of ornithine (the substrate for ODC), and depletion of putrescine (the product of ODC), consistent with direct inhibition of ODC by the drug (Fig. 2). The only other metabolites to consistently show marked changes in abundance were the acetylated forms of ornithine and putrescine. This study also demonstrated a significant depletion of spermidine, the downstream product of the polyamine pathway. Interestingly, only minor down-regulation was observed for trypanothione, the spermidine-bis-glutathione conjugate responsible for antioxidant activity in trypanosomatids, suggesting that the trypanocidal action of eflornithine is more likely related to polyamine depletion than disruption of the antioxidant defence system. Additional metabolite changes were detected after 48 h incubation with higher drug concentrations, highlighting the importance of metabolomic measurements across a dose-range and time-course to delineate direct drug mechanisms from downstream metabolic responses that may include non-specific cell death pathways (Vincent *et al.* 2012).

Metabolomic analysis of nifurtimox-treated cells revealed a complex pattern of metabolic perturbations, which could not be interpreted as inhibition of a single metabolic enzyme (Vincent *et al.* 2012). Altered abundances of nucleotide and glycolytic metabolites were consistent with previous hypotheses that implicate nucleic acid interactions and oxidative stress as likely mediators of nifurtimox activity. One advantage of the untargeted approach was the detection of nifurtimox metabolites that support the likely activation mechanism by *T. brucei* type-1 nitroreductase (Hall *et al.* 2011). In contrast to previous hypotheses, nifurtimox was found to lack synergistic activity with eflornithine, and metabolomics data support this lack of synergism (Vincent *et al.* 2012).

The application of metabolomics to experimental trypanocidal compounds can provide a method to elucidate attractive drug targets for future drug discovery. The impact of fluorinated pyrimidines on *T. brucei* metabolism was recently described (Ali *et al.* 2013). 5-fluoro-2′-deoxyuridine resulted in specific accumulation of dUMP in wild-type cells, and minimal change in resistant cells, demonstrating inhibition of thymidylate synthase, most likely by the active metabolite, 5-fluoro-dUMP. In contrast, 5-fluorouracil and 5-fluoroorotic acid were incorporated into the uracil nucleotide pool and then into parasite RNA, revealing a distinct mode of action for the fluoropyrimidines compared to the fluorodeoxynucleoside (Ali *et al.* 2013). The metabolomics platform has recently been applied to a variety of currently used and experimental trypanocidal drugs and work is following up on potential modes of action identified in this way.

### Antileishmania compounds

*Leishmania* spp. are trypanosomatid parasites responsible for a spectrum of diseases that include visceral, cutaneous and mucocutaneous manifestations of leishmaniasis. The infection is transmitted by sand-fly bite, and parasites develop mostly within macrophages of the mammalian host (Naderer and McConville, 2008). Treatment options for leishmaniasis are severely limited, and the commonly used treatments, pentavalent antimonials, amphotericin B, miltefosine and paromomycin, have poor safety, efficacy and pharmacokinetic profiles (Barrett and Croft, 2012). The mode of action of paromomycin has been investigated by proteomics and, like other aminoglycosides, appears to act by inhibition of protein synthesis (Chawla *et al.* 2011). Amphotericin B and miltefosine are thought to interact with sterols and phospholipid metabolism, respectively (Lux *et al.* 2000; Ouellette *et al.* 2004; Singh *et al.* 2012). It is expected that metabolomic or lipidomic studies would be able to confirm the impact of these drugs on lipid metabolism pathways to further characterize the modes of action and resistance.

The mode of action of antimonial compounds is a matter of debate, and is thought to be mediated primarily by oxidative stress. A metabolomics approach using CE-MS demonstrated significant perturbation of sulphur-containing amino acids and polyamine pathway metabolites following treatment of *Leishmania infantum* promastigotes with Sb(III) (Canuto *et al.* 2012). This finding is consistent with depletion of trypanothione, the main metabolite responsible for redox homeostasis in trypanosomatids. The mode of resistance to antimonials has also been studied by metabolomics with LC-MS, which revealed significant alterations in the membrane lipid composition of resistant *L. donovani* field isolates (t'Kindt *et al.* 2010*a*).

### Antimalarial compounds

*Plasmodium* spp. are the infectious agents responsible for malaria, with *P. falciparum* and *P. vivax* the most common causative agents of human disease. *Plasmodium* parasites undergo a complex life-cycle, and are spread by the bite of an infectious mosquito, with the symptomatic phase of disease occurring during replication of asexual parasites within red blood cells of the human host. Numerous drugs are approved for treatment of malaria, although resistance has emerged to most antimalarials (Dondorp *et al.* 2009; White, 2012). Artemisinin combination therapies (ACTs) are currently recommended due to their potent activity and limited evidence of clinical resistance to date (Anthony *et al.* 2012; Meshnick, 2012). Nevertheless, new antimalarials are urgently required, as ACTs suffer from emerging resistance, high cost and a pharmacokinetic mismatch between short-acting artemisinins and long-acting partner drugs, usually quinolines (Nosten and White, 2007; Anthony *et al.* 2012). Artemisinins are thought to act by production of free radicals following exposure of the peroxide pharmacophore to Fe(II) iron and/or haem, which is released as a by-product of haemoglobin digestion within the parasite (O'Neill *et al.* 2010). The quinoline antimalarials are also thought to act by interaction with intraparasitic haem, and preventing its detoxification (Loria *et al.* 1999). However, the precise mode of action of most antimalarials remains a matter of debate (Olliaro, 2001; O'Neill *et al.* 2010). The antifolate antimalarials are known to act by inhibition of DHFR and dihydropteroate synthase (DHPS), but the clinical application of these antifolates is limited by widespread resistance. Nevertheless, characterization of the target enzyme, DHFR, from wild-type and resistant parasites, has lead to the design of novel antimalarial leads that exhibit potent activity against the resistant enzyme (Yuthavong *et al.* 2012). This demonstrates the potential benefit of understanding the targets of current drugs as a paradigm for new drug discovery.

Atovaquone is another antimalarial with a known mode of action, inhibition of the cytochrome bc_1_ complex in the mitochondrial electron transport chain. This results in loss of the mitochondrial membrane potential and subsequent disruption of pyrimidine synthesis by indirect inhibition of the essential enzyme dihydroorotate dehydrogenase (DHODH). The mode of action of atovaquone has been confirmed by a targeted metabolomics study of *P. falciparum*, which demonstrated significant accumulation of dihydroorotate, the substrate of DHODH, and its precursor, carbamoyl-L-aspartate (Biagini *et al.* 2012). The mode of action for the novel quinolone, CK-2-68, was also confirmed by observation of the same metabolic response (Biagini *et al.* 2012).

Several compounds are currently under development for the treatment of malaria, and metabolomics studies may play an important role in defining the mode of action of new antimalarials. Fosmidomycin is an antibiotic that acts on *Plasmodium* parasites by inhibition of the essential non-mevalonate pathway of isoprenoid biosynthesis. A targeted metabolic profiling study of intermediates in the non-mevalonate isoprenoid synthesis pathway confirmed deoxyxylulose phosphate reductoisomerase (DXR) as the molecular target of the drug, and surprisingly, identified a second target in the pathway, methylerythritol phosphate cytidyltransferase (IspD) (Zhang *et al.* 2011). The accumulation of all metabolites upstream of IspD suggests that inhibition of this enzyme, either directly by fosmidomycin or by accumulation of 2-C-methylerythrose 4-phosphate, is the primary mode of isoprenoid depletion in *P. falciparum* (Zhang *et al.* 2011).

The response of *P. falciparum in vitro* cultures to polyamine pathway inhibitors, eflornithine and MDL73811, was investigated by targeted metabolomics combined with transcriptomics and proteomics. Polyamine depletion was confirmed as the primary mode of action, and unexpected accumulation of glutamate metabolites could be explained by transcriptional upregulation of ornithine aminotransferase in response to ornithine accumulation (van Brummelen *et al.* 2009).

## FUTURE DIRECTIONS

The suitability of metabolomics technology for determination of the mode of action of antiprotozoal compounds has been clearly demonstrated (Table 1). Further developments in technology will be necessary in order to expand the application of this approach efficiently to all the currently available antiprotozoal drugs, and the hundreds of compounds that display antiprotozoal activity *in vitro*. Whilst improvements in analytical hardware are expected to continue to expand metabolite coverage and increase sensitivity, the major issue limiting the accessibility of high-throughput metabolomics is data analysis. This situation is rapidly improving as evidenced by many recent advances in freely available data analysis software and web servers. However, it is expected that automated identification of metabolites will remain a significant bottleneck for untargeted metabolomics for the foreseeable future (Dunn *et al.* 2012).

**Table 1.** Examples of applications of metabolomics to determine the mode of action of drugs and other compounds with antimicrobial activity

Organism	Test compound	Metabolomics approach	Target identified	Reference
*T. brucei*	Eflornithine	Untargeted LC-MS (HILIC-Orbitrap)	Ornithine decarboxylase	(Vincent *et al.* 2012)
*T. brucei*	Nifurtimox	Untargeted LC-MS (HILIC-Orbitrap)	Complex: involves nucleotides, oxidative stress and an active metabolite	(Vincent *et al.* 2012)
*T. brucei*	5-fluoro-2′-deoxyuridine	Untargeted LC-MS (HILIC-Orbitrap)	Thymidylate synthase inhibited by active metabolite	(Ali *et al.* 2013)
*T. brucei*	5-fluoroorotate and 5-fluorouracil	Untargeted LC-MS (HILIC-Orbitrap)	RNA modification (fluorinated uracil residues)	(Ali *et al.* 2013)
*L. infantum*	Antimony (III)	Untargeted CE-MS (CE-TOF)	Oxidative stress (sulphur amino acids and polyamines)	(Canuto *et al.* 2012)
*P. falciparum*	Atovaquone and CK-2-68	Targeted LC-MS (HILIC-QQQ)	Dihydroorotate dehydrogenase (via mitochondrial electron transport chain)	(Biagini *et al.* 2012)
*P. falciparum*	Fosmidomycin	Targeted LC-MS (ion paired reversed-phase – QQQ)	Methylerythritol phosphate cytidyltransferase (IspD) and deoxyxylulose phosphate reductoisomerase (DXR)	(Zhang *et al.* 2011)
*P. falciparum*	Eflornithine with MDL73811	Targeted LC-MS (HILIC-QQQ)	Polyamine synthesis	(van Brummelen *et al.* 2009)
*Staphylococcus aureus*	Triphenylbismuth dichloride	Targeted ^1^H NMR	Pyruvate dehydrogenase	(Birkenstock *et al.* 2012)
*S. aureus*	Guanidinomethylbenzoates and guanidinobenzamides	Untargeted GC-MS	Classified as having similar mechanism to clindamycin	(Liu *et al.* 2009)
*Escherichia coli*	Trimethoprim	Targeted LC-MS (HILIC-QQQ)	Dihydrofolate reducatase and folylpoly-*γ*-glutamate synthetase	(Kwon *et al.* 2008)
*E. coli Pseudomonas aeruginosa*	11 common antibiotics	Targeted CE-UV	Classification of mechanisms by correlation analysis	(Gao *et al.* 2007)
*Mycobacterium smegmatis*	12 drugs and 3 chemical leads	Untargeted ^1^H NMR	Classification of mechanisms by multivariate analysis	(Halouska *et al.* 2012)
*M. smegmatis*	D-cycloserine	Untargeted ^1^H NMR	D-alanine-D-alanine ligase	(Halouska *et al.* 2007)

The ability to relate changes in metabolite abundance to drug action remains largely dependent on *a priori* knowledge of metabolic pathways within the parasite. Unfortunately, many aspects of parasite metabolism have not been fully elucidated, and often rely on bioinformatic reconstructions of metabolic pathways based on genomic homology with other organisms. Many parasite genes are not annotated, or annotated as hypothetical proteins, and it is likely that some drugs will cause perturbations in metabolites from uncharacterized pathways. In these cases, untargeted metabolomics studies of drug action are likely to provide significant new discoveries in basic parasite biochemistry, as has already been demonstrated for polyamine acetylation (Vincent *et al.* 2012) and pyrimidine metabolism (Ali *et al.* 2013) in *T. brucei*, and the two-step DXR reaction in *P. falciparum* isoprenoid biosynthesis (Zhang *et al.* 2011).

The major advantage of untargeted metabolomics is the ability to identify a drug-mediated response at the biochemical level with no prior knowledge of the mode of action of a particular antiprotozoal compound. This method can be used to classify compounds according to their mode of action based on a metabolic signature and, in many cases, highlight the specific metabolic enzyme or pathway responsible for antiprotozoal activity. The primary limitation of this approach is that some drugs do not act by disruption of metabolic pathways. In addition, drugs may induce non-specific metabolic changes in response to stress, or as secondary responses to the initial drug action. Targeted metabolomics approaches are often required in these circumstances to provide a detailed characterization of the temporal and dose-dependent biochemical effects of drugs. Additional studies, such as transcriptomics, may also be required to delineate primary and secondary metabolic responses to drug treatment (van Brummelen *et al.* 2009). Generally, measurements of RNA or protein levels are unlikely to reveal the primary targets of drugs that inhibit metabolic enzymes, particularly in trypanosomatid parasites that exhibit polycistronic transcription. Nevertheless, transcriptomic and proteomic approaches are likely to detect the secondary cellular responses to drug treatment, which may be relevant to determination of potential resistance mechanisms. A systems biology approach, that combines metabolomics with other functional genomics tools, is a promising avenue to obtain comprehensive descriptions of the modes of action and resistance for antimicrobial drugs. Metabolomics may also be combined with chemical biology (e.g. protein or ligand microarrays (MacBeath *et al.* 1999) and chemoinformatic methods (e.g. similarity ensemble approach - Keiser *et al.* 2010) to improve identification of unknown drug targets in an untargeted manner.

Metabolomics methods offer effective tools for the identification and characterization of mechanisms of drug action, which should optimize drug discovery by allowing detailed pre-clinical pharmacokinetic/pharmocodynamic descriptions of antiprotozoal drugs, and have the potential to translate into clinical studies to monitor both host and parasite responses to drug treatment (Beyoğlu and Idle, 2013).
